# Role of oxidative stress‐induced ferroptosis in cancer therapy

**DOI:** 10.1111/jcmm.18399

**Published:** 2024-05-17

**Authors:** Keqing Li, Chengjiang Fan, Jianing Chen, Xin Xu, Chuwei Lu, Hanjie Shao, Yang Xi

**Affiliations:** ^1^ Department of Biochemistry and Molecular Biology, Zhejiang Key Laboratory of Pathophysiology, School of Basic Medical Sciences, Health Science Center Ningbo University Ningbo China

**Keywords:** ferroptosis, molecular pathways, oxidative stress, reactive oxygen species, targeted drugs, tumours

## Abstract

Ferroptosis is a distinct mode of cell death, distinguishing itself from typical apoptosis by its reliance on the accumulation of iron ions and lipid peroxides. Cells manifest an imbalance between oxidative stress and antioxidant equilibrium during certain pathological contexts, such as tumours, resulting in oxidative stress. Notably, recent investigations propose that heightened intracellular reactive oxygen species (ROS) due to oxidative stress can heighten cellular susceptibility to ferroptosis inducers or expedite the onset of ferroptosis. Consequently, comprehending role of ROS in the initiation of ferroptosis has significance in elucidating disorders related to oxidative stress. Moreover, an exhaustive exploration into the mechanism and control of ferroptosis might offer novel targets for addressing specific tumour types. Within this context, our review delves into recent fundamental pathways and the molecular foundation of ferroptosis. Four classical ferroptotic molecular pathways are well characterized, namely, glutathione peroxidase 4‐centred molecular pathway, nuclear factor erythroid 2‐related factor 2 molecular pathway, mitochondrial molecular pathway, and mTOR‐dependent autophagy pathway. Furthermore, we seek to elucidate the regulatory contributions enacted by ROS. Additionally, we provide an overview of targeted medications targeting four molecular pathways implicated in ferroptosis and their potential clinical applications. Here, we review the role of ROS and oxidative stress in ferroptosis, and we discuss opportunities to use ferroptosis as a new strategy for cancer therapy and point out the current challenges persisting within the domain of ROS‐regulated anticancer drug research and development.

## 1 INTRODUCTION

Oxidative stress, indicated by high levels of reactive oxygen species (ROS), exists in cell metabolism and several diseases, including tumours.^1^, ^2^ ROS possesses the capability to modify the activity of transcription factors and hold a significant role in governing cellular proliferation and differentiation. Nonetheless, the irregular regulation of ROS generation coupled with their elusion from antioxidant defences leads to oxidative stress, resulting in harm to DNA, proteins and lipids. This, in turn, can trigger processes like carcinogenesis or cell death. Contemporary research has distinguished various modes of cell demise, encompassing apoptosis, autophagy, necrotizing apoptosis and ferroptosis, among others. Out of these, ferroptosis emerges as a recently revealed form of programmed cell death, initially introduced by Stockwell et al. in 2012.^3^ The induction of ferroptosis depends on iron ions, whereas the presence of unstable iron (Fe^2+^) significantly increases the risk of oxidative stress due to the formation of hydroxyl radicals through the Fenton reaction to develop oxidative damage, especially the peroxidation of membrane lipids.

However, the regulatory mechanism underlying oxidative stress and ferroptosis is drawing increasing attention. This article describes the recent progress in the mechanism of ferroptosis to explain the function of ROS in it.

### 1.1 Oxidative stress

Oxidative stress is often defined as a state of relative excess of ROS compared with antioxidants.^4^ ROS is a general term for oxygen metabolites and their derivatives that are chemically active after molecular oxygen is reduced by a single electron. These mainly include superoxide anions (O_2_–), hydrogen peroxide (H_2_O_2_), hydroxyl radicals (HO), molecular oxygen (O_2_) and hypochlorite (HOCl). Depending on the source of ROS generation, ROS can be divided into two types, namely, endogenous and exogenous. Exogenous ROS is formed after stimulation by factors, including radiation, drugs, high pressure and others. Endogenous ROS are produced by mitochondria, peroxidase and endoplasmic reticulum. About 90% of endogenous ROS originate from complex I and complex III in the mitochondrial electron transport chain (ETC) as a by‐product of cellular metabolism. Others, including the cytochrome P450 pathway and oxidative phosphorylation pathway, can also produce ROS.^5^ As a strong oxidizing oxygen fragment, ROS can cause damage to DNA, RNA, proteins, lipids and so on without removal.^6^ On one hand, ROS promote the activation of transcription factors and play a positive role in cell proliferation and differentiation; on the other hand, when ROS formation is maladjusted and exceeds antioxidant defence, oxidative stress will occur and induce cell damage or carcinogenesis.

Oxidative stress may lead to the occurrence and progression of many diseases, including cancer with varying degrees of importance.^7^ For example, oxidative stress is also associated with several neurological diseases, such as Parkinson's disease, Alzheimer's disease^8^ and vascular dementia.^9^ It was found that in addition to the neuroprotective effect of physiological dose nitric oxygen,^10^ cell stress response and vitagenes are new targets for the treatment and intervention of neurodegenerative diseases, in which vitagenes is the gene involved in maintaining cell homeostasis under stress conditions.^8^ Curcumin is one of the representative drugs developed.^11^ Oxidative stress can drive and promote cancer by chromosomal abnormalities and oncogene activation^12^, ^13^ through various modifications of DNA structure, such as base and sugar damage, DNA–protein cross‐links, strand breaks and non‐base site.^12^, ^14^, ^15^ While cancer cells are under continuous oxidative stress, the basic level of ROS increases due to increased metabolism driven by abnormal cell growth, but cancer cells can adapt to maintain redox homeostasis through a variety of mechanisms. It is suggested that targeted tumour redox can amplify the oxidative stress within the tumour and further induce iron death, resulting in the destruction of tumour cells for cancer therapy.^16^

## 2 FERROPTOSIS

Ferroptosis is a recently discovered form of programmed cell death that is distinct from apoptosis, necrosis and autophagy. The process of ferroptosis depends on iron ions and is characterized by three basic features such as the utilization of redox‐active iron, loss of lipid peroxide (LOOH) repair capacity and the oxidation of polyunsaturated fatty acid (PUFA)‐containing membrane phospholipids.^17^ A growing body of evidence suggested that ferroptosis is associated with various human diseases, such as cancer, neurodegenerative diseases, infections and inflammation.^18^, ^19^, ^20^

**FIGURE 1:**
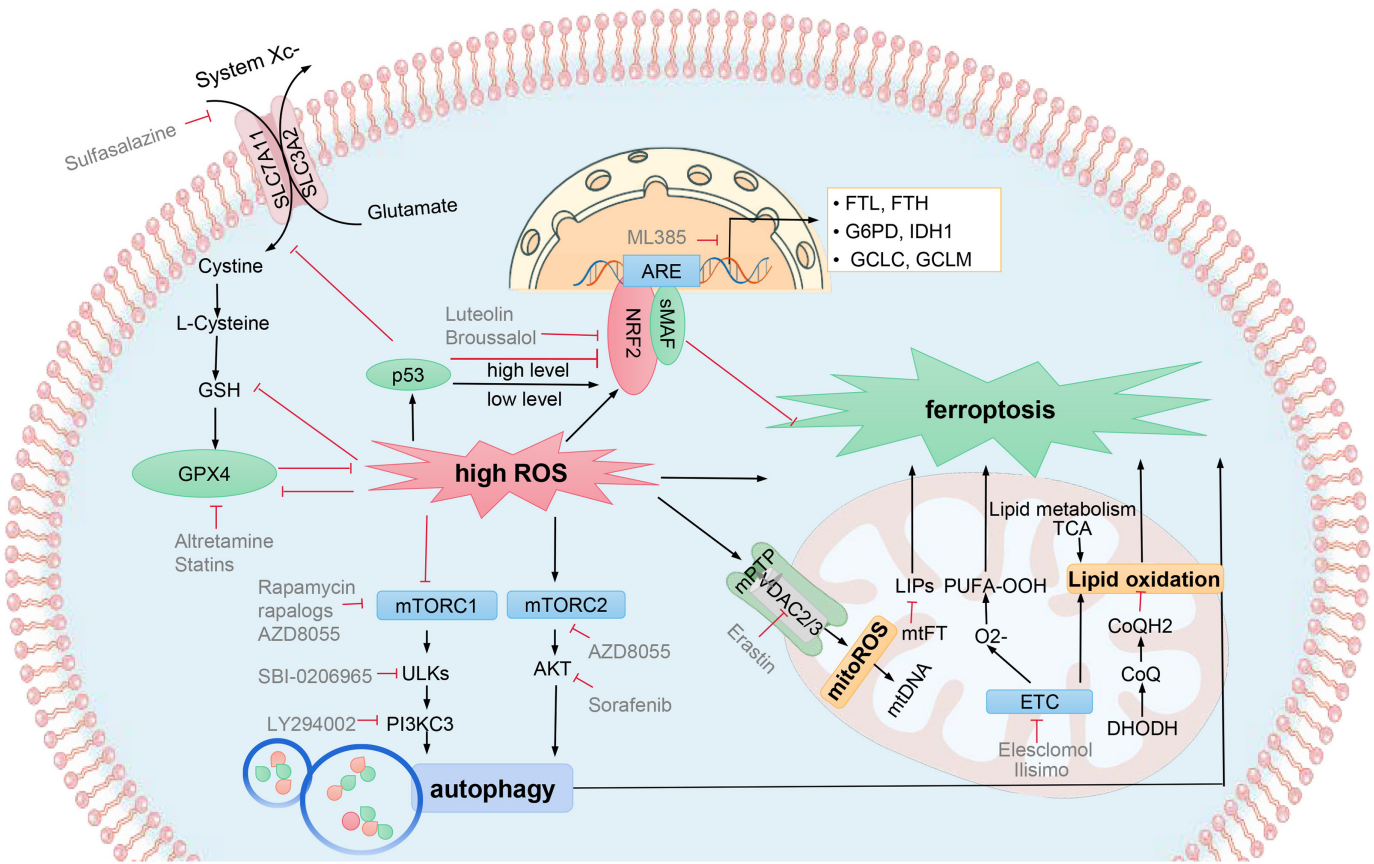
Molecular pathways governing the regulation of ferroptosis. Four pathways as glutathione peroxidase 4 (GPX4)‐centred pathway, nuclear factor erythroid 2‐related factor 2 (NRF2) molecular pathway, mitochondrial pathway and mTOR‐dependent autophagy pathway are included. The activity of GPX4 relies on glutathione (GSH) that is synthetized from cysteine and glutamate. High reactive oxygen species (ROS) activate p53 and indirectly affect GPX4 activity, which inhibits the transcription of SLC7A11, a key component of system Xc‐. Altretamine inhibits the activity of GPX4. Statins inhibit the biosynthesis of GPX4. Sulfasalazine is a class of effective system Xc‐ inhibitors. ROS increase NRF2, which translocates into the nucleus, forms a complex with small Maf (sMAF) and antioxidant response element (ARE), and activates target genes that regulate iron and GSH metabolism. Luteolin inhibits NRF2; ML385 binds to NRF2 and inhibits its target genes. Broussalol can enhance the degradation of NRF2. In mitochondrial lipid oxidation, PUFA‐OOA and labile iron pools contribute to ferroptosis, whereas mitochondrial ferritin (mtFT) and dihydroorotate dehydrogenase (DHODH) inhibit ferroptosis. High ROS open mitochondrial permeability transition pore (mPTP) to stimulate ROS leakage, resulting in lipid peroxidation and mitochondrial (mtDNA) damage. Elesclomol and Ilisimo are electron transport chain‐targeting compounds. Erastin can inhibit VDAC2/3. At high ROS level, mammalian target of rapamycin complex (mTORC) 1 is inhibited to activate the Unc‐51‐like kinase (ULKs) complex, which stimulates the activation of the PI3KC3 complex and in turn promotes autophagy‐dependent ferroptosis. The mTORC2 promotes the activation of protein kinase B (AKT) and autophagy to contribute to ferroptosis. Rapamycin and rapalogs are allosteric selective inhibitors of mTORC1. AZD8055 inhibits two mTOR complexes. LY294002 inhibits the activity of the PI3K complex. SBI‐0206965 and sorafenib targets ULK1 and AKT, respectively.

Ferroptosis is actively triggered by specialized pro‐death molecular mechanisms, and four classical ferroptotic molecular pathways are well characterized, namely, glutathione peroxidase 4 (GPX4)‐centred molecular pathway, nuclear factor erythroid 2‐related factor 2 (NRF2) molecular pathway, mitochondrial molecular pathway, and mTOR‐dependent autophagy pathway (Figure 1). In the process of tumorigenesis, ferroptosis induced by oxidative stress can promote or inhibit tumour, which depends on the release of damage‐related molecular model in tumour microenvironment and the activation of immune response induced by ferroptosis.^21^, ^22^ Ferroptotic injury can trigger inflammation‐related immunosuppression in tumour microenvironment, which is beneficial to tumour growth.^23^ However, in most cases, induced ferroptosis may be a useful tumour treatment strategy. Ferroptosis induced by oxidative stress helps to inhibit tumour growth and increase chemosensitivity.

### 2.1 GPX4‐centred molecular pathway

GPX4 is a key enzyme in the GPXs family and is involved in the reduction of toxic peroxides. GPX4 prevents the toxicity of lipid peroxides and maintains the equilibrium of the membrane lipid bilayer.^24^ Therefore, a decrease in the activity of GPX4 will increase the sensitivity of cells to ferroptosis.^25^ Also, the activity of GPX4 closely depends on glutathione (GSH)^26^ synthesized from cysteine and glutamate, which has intracellular concentrations and is fine‐tuned by the amino acid antiporter system Xc‐.^27^ Under oxidative conditions, ROS regulate GPX4 expression in two ways: one in which the continuous accumulation of ROS decreases the level of GSH and the expression of GPX4,^28^ and the other in which ROS activate p53 and indirectly affect the activity of GPX4. It has been reported that high levels of H_2_O_2_ can activate p53,^29^ which inhibits the transcription of the cysteine/glutamate antiporter SLC7A11, a key component of system Xc‐.^30^, ^31^

### 2.2 NRF2 molecular pathway

The NRF2 is a core protein of the antioxidant stress response in the cytoprotective pathway. NRF2 regulates ferroptosis by three methods. First, NRF2 induces the expression of genes encoding the components of the ferritin complex, namely, the ferritin light polypeptides and heavy polypeptides.^32^ The ferritin complex oxidizes Fe^2+^ to Fe^3+^ and stores it within its structure, thus making it unavailable for the Fenton reaction and inhibiting ferroptosis.^33^ Second, NRF2 supports the production of NADPH, which is an obligatory cofactor for antioxidant systems, by positively regulating the principal NADPH‐generating enzymes such as glucose‐6‐phosphate dehydrogenase and isocitrate dehydrogenase 1.^34^ Third, NRF2 tightly regulates GSH levels by directly controlling the expression of the two subunits constituting the glutamate–cysteine ligase (GCL) complex, namely, the catalytic subunit (GCLC) and the modifier subunit (GCLM).^35^ GCL facilitates the amalgamation of glutamate with cysteine, constituting the pivotal pace‐determining phase within the GSH production process. Upon exposure to ROS, there is an elevation in the abundance of NRF2 protein. Subsequently, this protein relocates to the cell nucleus, where it associates with the small Maf protein. This complex then affixes to the antioxidant response element (ARE), thereby prompting the initiation of transcription for target genes. This regulatory sequence is instrumental in preserving cellular redox equilibrium and restraining the onset of ferroptosis.^36^

### 2.3 Mitochondrial molecular pathway

Mitochondria play facilitating or inhibiting roles in ferroptosis. Their facilitating roles include the following: (1) Acting as a major source of cytosolic lipid peroxide through the mitochondrial tricarboxylic acid cycle and the ETC, which contributes to cysteine deprivation‐induced ferroptosis.^37^, ^38^ Cells with disrupted glycolysis enhance oxidative phosphorylation from mitochondria, leading to ferroptosis.^39^ (2) Causing electron leakage from ETC complexes I and III to generate O_2_^−^, which eventually forms polyunsaturated fatty acid hydroperoxides (PUFA‐OOH).^37^ (3) Inducing mitochondrial lipid metabolism, which may be a key step in the execution of ferroptosis by providing specific lipid precursors for lipid oxidation.^40^ During injury due to oxidative stress, ROS trigger the formation and opening of the mitochondrial permeability transition pore (mPTP), which further stimulates the production of ROS related to the opening of mPTP. This phenomenon is called the ‘ROS‐induced ROS release’.^41^ After the mitochondrial ROS are accumulated, they can react with PUFA on the mitochondrial membrane, resulting in lipid peroxidation and mitochondrial DNA (mtDNA) damage, which then leads to defective ETC complex mtDNA‐coding subunits.^42^

On the other hand, the inhibitory roles of mitochondria in ferroptosis include: (1) Mitochondrial ferritin (mtFT) acts as an iron‐storage protein that regulates iron metabolism. The overexpression of mtFT has been reported to inhibit erastin‐induced ferroptosis by increasing the amount of iron in storage and decreasing the levels of cellular labile iron pools (LIPs) and ROS.^43^ (2) Dihydrolactate dehydrogenase‐mediated ferroptosis defence: dihydroorotate dehydrogenase is a mitochondrial enzyme that reduces the coenzyme Q (CoQ) to its hydroquinone form of coenzyme Q (CoQH_2_).^44^ CoQH_2_ acts as a free‐radical capture antioxidant (RTA), quenching the free radicals from lipid peroxidation in the inner mitochondrial membrane and thus inhibiting ferroptosis.

### 2.4 The mTOR‐dependent autophagy pathway

Ferroptosis depends on autophagy,^45^, ^46^ a lysosomal degradation pathway responsible for recycling damaged cellular components under survival stress. Autophagy contributes to ferroptosis by breaking down ferritin,^47^ an iron‐storage protein, accompanied by increased iron levels and lipid ROS. Distinct types of autophagy orchestrate the complex ferroptotic response through direct or indirect regulation of iron accumulation or lipid peroxidation.^48^

In mammalian cells, autophagy initiation involves the inactivation of mammalian target of rapamycin (mTOR), a serine/threonine kinase involved in various biological processes.^49^ mTOR forms two complexes with distinct functions: mTOR complex 1 (mTORC1) and mTORC2. At moderate ROS levels, mTORC1 and mTORC2 inhibit autophagy‐dependent ferroptosis.

However, at high ROS levels or under stress conditions, mTORC1 inhibition triggers the activation of the Unc‐51‐like kinase (ULKs) complex, leading to phagocytic nucleation. This activation further stimulates the phosphatidylinositol 3‐kinase catalytic subunit type 3 (PI3KC3) complex. Active PI3KC3 promotes phagocyte formation and expansion. These elongated phagocytes develop into autophagosomes,^50^ promoting autophagy‐dependent ferroptosis. Moreover, mTORC2 contributes to protein kinase B (Akt) activation by phosphorylating Akt at S473 (pAkt). It also supports autophagy, contributing to ferroptosisprogression.^51^, ^52^

## 3 ASSOCIATION BETWEEN ROS AND FERROPTOSIS IN CANCER THERAPY

The occurrence and development of cancer is a complex multi‐stage process. In the early stage of cancer, damage to nuclear or mtDNA and metabolic adaptation induced by ROS play important roles in the occurrence and promotion of cancer. ROS are maintained at low to moderate levels and act as signal transducers to activate the proliferation, migration, invasion, angiogenesis and drug resistance.^53^, ^54^, ^55^ At this time, ferroptotic damage can trigger inflammation‐related immunosuppression in the tumour microenvironment, which supports tumour growth.^22^ However, in the advanced stages of cancer, ROS are maintained at relatively high levels^56^ and ROS‐induced ferroptosis helps inhibit tumour growth and increase chemosensitivity. Through the experimental studies, we summarized the representative drugs and the corresponding targeting and applications in Table 1, it has been found that ferroptosis can reduce drug resistance in several types of cancer cells during ongoing cancer treatment, which provides a new direction for cancer treatment.^70^ As displayed in Figure 1, new avenues for the development of novel therapeutics targeting ferroptosis for the treatment of cancer have been developed and summarized.^71^, ^72^

**TABLE 1:** Summary of representative drugs in cancer therapy targeting ferroptosis.

Drug	Mechanism	Application model	Details and analyses of treatment	Reference
Altretamine	Inhibits GPX4 activity	The diffuse large B‐cell lymphoma U‐2932 cells	Altretamine‐treated U‐2932 cells displayed a significant reduction of phosphatidylcholine hydroperoxide, a specific substrate for GPX4, and improved cell death	^57^
Fluvastatin	Inhibits the biosynthesis of GPX4	Ductal carcinoma or breast cancer	Proliferation of high grade tumours decreased by a median of 7.2 percentage points (*p* = 0.008) with fluvastatin treatment over a treatment period that ranged from 21 to 50 days	58, 59
Luteolin	Inhibits NRF2 to deplete GSH	Tumour‐bearing mice with HCT116 cells	Combined luteolin and oxaliplatin treatment significantly inhibited tumour growth of tumour cells in xenograft mice with an average 4.26‐fold increase versus the untreated 8.28‐fold	60, 61
ML385	Binds to NRF2 to inhibit its target genes	A549 and H460 cells in athymic nude mice	Increased twofold high platinum level in A549 and H460 tumours in xenograft mice treated with carboplatin alone or ML385 in combination with carboplatin	^62^
Elesclomol	ETC‐targeting compound	Melanoma cells	Through chelating copper ions, elesclomol promotes mitochondrial ROS by destroying ETC, induces oxidative stress and cell death	^63^
Erastin	Targets VDAC2/3 to increase ROS	Human glioblastoma U87MG and A172 cells	Erastin can open VDAC2/3 channel to increase the mitochondrial metabolism and ROS levels resulting in oxidative stress and enhance the sensitivity of glioblastoma cells to temozolomide	64, 65
Rapamycin	Inhibitor of mTORC1 to regulate autophagy	Neuroblastoma cells	Rapamycin treatment greatly increased the amounts of autophagosomes and autolysosomes in neuroblastoma cells by decreasing the mTOR and p‐mTOR levels	66, 67
AZD8055	Inhibits two mTOR complexes	Hepatocellular carcinoma cell line HepG2	AZD8055 inhibits the phosphorylation of mTORC1 substrates p70S6K and 4E‐BP1 as well as phosphorylation of the mTORC2 substrate AKT and downstream proteins	68, 69

Abbreviations: ETC, electron transport chain; GPX4, glutathione peroxidase 4; GSH, glutathione; mTOR, mammalian target of rapamycin; mTORC, mammalian target of rapamycin complex; NRF2, nuclear factor erythroid 2‐related factor 2.

### 3.1 Targeting the GPX4 molecular pathway

Cancer drugs inhibiting the activity of GPX4 have achieved great results. Altretamine is an alkylated antineoplastic drug approved by Food and Drugs Administration (FDA) for the treatment of ovarian cancer. It can inhibit GPX4 in vitro and effectively kill the diffuse large U‐2932 B‐cell lymphoma cells.^57^ Statins inhibit the biosynthesis of GPX4 by inhibiting selenocysteine tRNA, a special transporter of amino acids in the active centre of GPX4.^58^, ^59^ Data from clinical trials reveal that fluvastatin has a stronger antiproliferative effect on breast cancer cells overexpressing 3‐hydroxy‐3‐methylglutaryl CoA reductase (HMGCR) than the traditional antineoplastic drug tamoxifen.^58^ Furthermore, statins can be employed synergistically with other treatments to enhance the anticancer impact. For instance, individuals afflicted by liver cancer experience improved outcomes with the combination of licatine and transcatheter hepatic arterial chemoembolization (TACE), surpassing the effects of TACE in isolation. In the case of multiple myeloma, treatment involves a regimen encompassing thalidomide, dexamethasone and lovastatin, leading to extended overall survival and progression‐free survival. Another approach entails the targeting of system Xc‐. An exemplar is sulfasalazine, categorized as a potent system Xc‐ inhibitor.^73^, ^74^ Sulfasalazine can effectively inhibit the system Xc‐ activity of CD133‐positive hepatoma cells, weaken the ROS defence system and enhance the proliferation inhibition activity of cisplatin and DOX in CD133‐positive hepatoma cells.^75^

### 3.2 Targeting the NRF2 molecular pathway

NRF2 expression level is high in cancer cells and also cancer stem cells, suggesting that drugs targeting NRF2 and its downstream antioxidant genes may play a centre role in the treatment of cancer.^76^ The flavonoid luteolin inhibits NRF2 at both mRNA and protein levels, causing a decrease in the binding of NRF2 to ARE, resulting in the depletion of GSH.^60^ Luteolin can significantly enhance the antitumor effects of oxaliplatin, cisplatin and DOX in drug‐resistant colon cancer cells.^61^ There are also drugs binding to NRF2, which directly inhibit the expression of its downstream target genes; for example, the probe molecule ML385. In NSCLC cells with highly activated NRF2, ML385 combined with carboplatin could produce a significant antitumor effect.^62^ Another strategy is to focus on enhancing the degradation of NRF2. Broussalol can effectively decrease the level of intracellular NRF2 by enhancing the ubiquitination and degradation of NRF2, and the NRF2‐dependent protective response is inhibited to enhance the effect of several tumour cell types, including lung cancer cells.^77^

### 3.3 Targeting the mitochondrial molecular pathway

Significant progress has been made in the development of drugs targeting ETC. Elesclomol is an ETC‐targeting compound that was approved by the United States FDA in 2008 as an orphan drug for the treatment of metastatic melanoma. Clinical trials on metastatic solid tumours and acute myeloid leukaemia (NCT00808418 and NCT01280786) are ongoing in the United States. Elesclomol is transformed into its active form by chelating copper ions in vivo, and this complex promotes the production of mitochondrial ROS by destroying ETC, induces oxidative stress and eventually leads to the death of melanoma cells. This effect is partially reversed by the mitochondria‐targeting antioxidant Mito‐Tempo.^63^ Erastin can specifically inhibit the function of tubulin on the voltage‐dependent anion channel (VDAC) proteins on the outer membrane of mitochondrial VDAC2/3, prevent the blockage of VDAC2/3 by cytoplasmic free tubulin and open VDAC2/3.^64^ The opening of VDAC2/3 can increase the mitochondrial metabolism, decrease glycolysis and increase the ROS levels, resulting in oxidative stress. Erastin can enhance the sensitivity of lung cancer cells to cisplatin, rhabdomyosarcoma cells to doxorubicin and actinomycin D and glioblastoma cells to temozolomide.^65^, ^78^ Moreover, erastin can also eliminate drug resistance in various chemotherapy‐resistant cells,^79^ enhancing its applicability as an anticancer drug.

### 3.4 Targeting the mTOR‐dependent autophagy pathway

The mTOR inhibitors play an important role in inducing tumour autophagic cell death (ACD). Rapamycin and its semisynthetic analogues (called rapalogs) are allosteric selective inhibitors of mTORC1 that affect their downstream targets, including the activation of autophagy.^66^, ^80^ Rapamycin has been shown to inhibit the proliferation of neuroblastoma,^67^ lung cancer^81^ and osteosarcoma^82^ and induce ACD. The rapalog temsirolimus inhibits tumour growth in adenoid cystic carcinoma in vitro.^83^ AZD8055 inhibits two mTOR complexes, inhibits tumour growth^68^ and induces ACD in hepatocellular carcinoma (HCC) cell lines.^69^ It can also limit tumour growth by inducing apoptosis and cell cycle arrest.^84^ During the initiation and nucleation of autophagy, LY294002 inhibits the activity of the PI3K complex and has therapeutic effects on melanoma.^85^ SBI‐0206965 targets the ULK1 and AMP‐activated protein kinase (AMPK) and has therapeutic effects on renal cell carcinoma and neuroblastoma.^86^, ^87^ Sorafenib targets AKT and plays a role in the treatment of HCC.^88^

## 4 CONCLUSIONS

Ferroptosis triggered by ROS contributes to restraining tumour growth and enhancing chemosensitivity. Given the mounting recognition of ferroptosis activation as a fresh drug discovery focus, there is an expanding array of small molecular compounds identified for their capacity to directly or indirectly induce ferroptosis. This is achieved through targeting iron metabolism and lipid peroxidation. Notable strides have been achieved in cancer therapy. Nonetheless, challenges persist within the domain of ROS‐regulated anticancer drug research and development, warranting further investigation and optimization.

Of primary note is the relatively modest antitumor activity displayed by ROS. Typically, they are harnessed as chemotherapeutic sensitizers or adjuvant drugs in treatment. While augmenting ROS to cytotoxic levels holds promise for cancer cell eradication, this strategy inevitably entails systemic toxicity akin to traditional chemotherapy and radiotherapy. Additionally, ROS regulators tend to lack target specificity, raising concerns about potential adverse reactions during prolonged use. A pivotal question in cancer redox biology pertains to the feasibility of leveraging ROS for the precise elimination of tumour cells, without indiscriminately affecting normal cells. These limitations collectively hamper the clinical applicability of such drugs.

Subsequent research in this domain should not only emphasize the antitumor attributes of ROS regulators or their synergistic combinations with other agents but also encompass the development of additional ROS agents to fine‐tune drug selectivity towards their targets. Novel molecular probes capable of monitoring ROS with temporal and spatial precision will shed light on the intricate regulatory relationships between diverse redox pairs and their downstream effects on distinct subcellular organelles.

Further advancement in comprehending ROS‐associated pathways and their consequential functions is imperative for enhancing our grasp of the distinctive roles ROS play in normal and cancerous cells.

## AUTHOR CONTRIBUTIONS

**Keqing Li:** Conceptualization (equal); writing – original draft (equal). **Chengjiang Fan:** Resources (equal); validation (equal). **Jianing Chen:** Conceptualization (equal); writing – review and editing (equal). **Xin Xu:** Resources (equal). **Chuwei Lu:** Resources (equal). **Hanjie Shao:** Resources (equal). **Yang Xi:** Conceptualization (lead); funding acquisition (lead); supervision (lead); writing – review and editing (lead).

## CONFLICT OF INTEREST STATEMENT

The authors declare no conflict of interest.

## Data Availability

Data are available from the corresponding author as required.
